# Systemic administration of Follistatin288 increases muscle mass and reduces fat accumulation in mice

**DOI:** 10.1038/srep02441

**Published:** 2013-08-14

**Authors:** Samudra S. Gangopadhyay

**Affiliations:** 1 Section of Endocrinology, Diabetes, and Nutrition Department of Medicine Boston University School of Medicine 670 Albany Street, Boston, MA 02118; 2 Current address: Department of Urology, Boston Children's Hospital, 300 Longwood Avenue, Boston, MA 02115 and Department of Surgery, Harvard Medical School, Boston MA.

## Abstract

The present study describes the physiological response associated with daily subcutaneous injection of mice with recombinant follistatin288. This systemic administration of follistatin288 increases the follistatin levels in serum, indicating that the protein enters the circulation. The data suggest that a dose-dependent increase in body lean mass also occurs, together with an increase in muscle mass, possibly as a result of an increase in the size of the muscle fibers. After thirteen weeks of treatment, metabolic changes were observed; additionally, the switching of muscle fiber types was also apparent through myosin heavy chain remodeling, implying that changes are occurring at the molecular level. Furthermore, an increase in the muscle mass was associated with a significant decrease in the body fat mass. Overall, this study raises the possibility for the use of follistatin288 as an agent to treat muscle wasting diseases and/or to restrict fat accumulation by systemic administration of the protein.

The role of transforming growth factor-β (TGF-β)-mediated signaling has been well established in several essential cellular and developmental processes, including differentiation, migration, proliferation, survival and adult tissue homoeostasis^1^,^2^,^3^. TGF-β is a superfamily of cytokines that are ubiquitously expressed in a range of species, from worms and flies to mammals. Members of this superfamily function by binding specific cell surface receptors (type I & II), which, in turn, activate the Smad proteins. The activated Smad proteins undergo nuclear translocation and, together with other transcriptional co-activators and co-repressors, regulate the expression of downstream target genes^4^,^5^. In addition to the canonical Smad mediated pathway, TGF-β proteins also mediate other non-Smad pathways, including MAP Kinase, p53, PI3/Akt, JNK and NFκB pathways^6^,^7^. Furthermore, the diversity of TGF-β functions arises through its regulation at multiple levels, beginning at the ligand, the receptor and also the level of the transcriptional activation complex formation^3^,^8^. Within the TGF-β superfamily, the activity of the growth and differentiation factor (GDF) family proteins has drawn increasing attention. The GDF family was discovered to have possible therapeutic applications in the treatment of muscle wasting diseases or muscle loss conditions that are associated with other pathological conditions, including obesity and aging. In this regard, the discovery of GDF-8 (popularly known as myostatin) as a negative regulator of muscle growth raises the possibility of developing new targets to limit its function in the body, thereby facilitating muscle growth^9^,^10^. The use of multiple pharmacological inhibitors to block the activity of myostatin^11^,^12^,^13^,^14^, as well as genetic alteration studies^15^,^16^ in animals, is very inspiring, and several clinical trials targeting this pathway to treat muscle wasting are ongoing. However, the recent development of endogenous TGF-β inhibitory proteins provides new insight into the regulation of TGF-β function in muscle development. In this regard, Follistatin (FST), a potent myostatin antagonist, seems a good candidate with potential for use as a therapeutic agent. FST antagonizes myostatin activity by binding to it and also by interfering with the binding of myostatin to its receptor^17^,^18^,^19^, but *in vivo* studies indicate that myostatin may not be the only regulator of muscle mass and may not be the only target of FST^19^. Direct interaction between follistatin and myostatin has been established^17^ and inhibition of TGF-β signaling by follistatin has been reported^20^. The actual mechanism of action of FST is unclear, but the use of FST to stimulate muscle growth has been considered for therapeutic application^13^,^21^,^22^. In the present study, the strategy was to introduce recombinant FST288 into animals via daily subcutaneous injection. Continuous monitoring of the physiological response associated with the daily injection showed an increase in the lean mass in a dose-dependent manner, and by thirteen weeks, a significant increase in the muscle mass was observed. The results indicate that the increased muscle mass is caused by an increase in the average size of the muscle fiber. Moreover, a switch in the muscle fiber type was observed as a result of myosin heavy chain remodeling. The study is also significant as there was a concomitant loss of fat mass along with a gain of lean body mass, which is indicative of a healthy metabolic condition.

## Results

### Recombinant FST288 is biologically active

**Figure 1:**
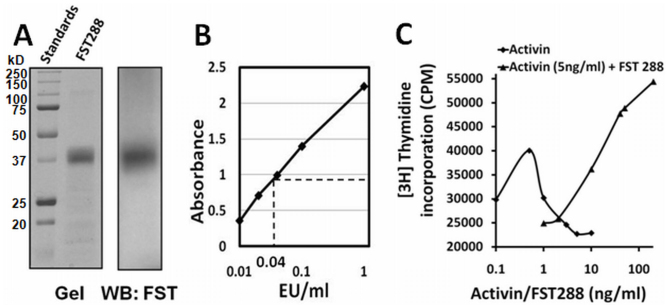
Recombinant FST288 is biologically active. (A) Coomassie blue stained full-length gel image of purified FST288 (left panel), and western blot probed with the anti-FST antibody (right panel). (B) Endotoxin in the protein preparation. The amount of endotoxin was determined (dotted line) from a standard curve in the assay. (C) Activin-induced inhibition of the MPC-11 cell proliferation is blocked by the addition of increasing amounts of recombinant FST288 in the presence of 5 ng/ml activin.

N-terminal His-tagged human FST288 was expressed in *E. coli* and purified by one-step purification using a HisPur cobalt column, yielding almost 90% pure protein, as determined from the Coomassie blue-stained gel image (figure 1A). The protein was then purified with a Detoxi-Gel column to remove bacterial endotoxins, which resulted in an approximately 25-fold decrease in the endotoxin level, as determined by the Toxin Sensor LAL endotoxin assay (GenScript, USA). The final endotoxin concentration in the protein preparation was approximately 0.04–0.06 E.U./ml (figure 1B); the protein can be considered as ‘endotoxin free' as this is a very low concentration, within the recommended safety levels (Study of McIntyre and Reinin, BD Biosciences). A cell proliferation analysis of a plasmacytoma cell line, MPC-11, was performed to test the biological activity of recombinant FST288^23^. Growth inhibition was observed in the presence of 0.1–10.0 ng/ml activin in the growth medium (figure 1C), as assessed by the decrease in ^3^H incorporation. However, the presence of increasing amounts of FST288 (1–200 ng/ml) in the growth medium blocked the growth-inhibitory effect of 5.0 ng/ml activin in a dose-dependent manner (figure 1C).

### The injected recombinant FST288 enters the systemic circulation

**Figure 2:**
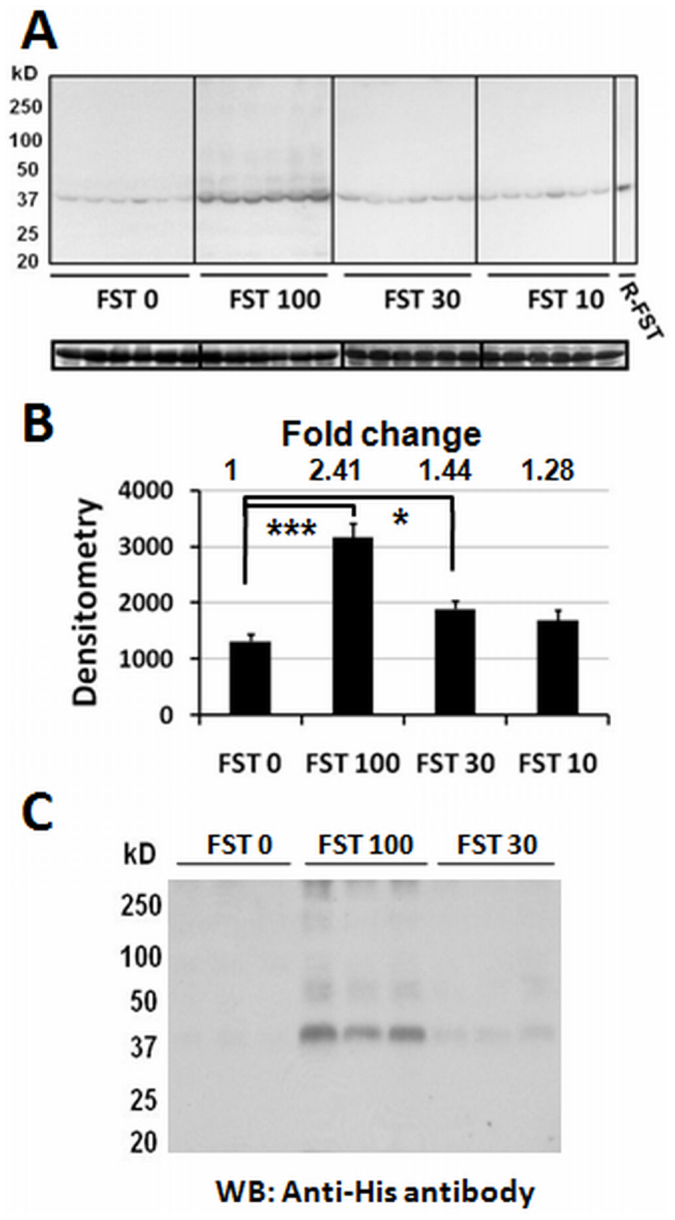
FST288 in the systemic circulation. (A) Western blot (full-length) of serum probed with the follistatin antibody (upper panel). Recombinant FST288, used as the control, is detected in the blot and labeled as R-FST. Bands of serum albumin, assessed as a loading control, are presented in the lower panel. (B) The densitometry of the average western blot signals from each group as obtained in A. The fold change with respect to the FST 0 group is presented above the bar. (C) Western blot (full-length) of serum from three animals of three groups, as indicated, probed with the anti-His antibody. * and *** indicate p < 0.05 and <0.001, respectively.

The level of follistatin in the serum of each animal was tested at the end of week 13: 0.2 μl of serum (diluted to 20 μl) was separated with a 12% SDS-PAGE gel, and the separated proteins were transferred to an Immobilon-Fl membrane (Millipore). Subsequent immunodetection with the anti-follistatin antibody (k-19) showed a single band with variable signal intensity between the different groups (figure 2A). The mobility of the protein band matches that of the recombinant purified protein loaded in the right lane. A densitometric analysis of the average signal intensity from each group shows that the FST 100 group has an approximately 2.41-fold higher follistatin level in the serum than the FST 0 control group. The densitometric analysis of the FST 30 & 10 groups does not show significant differences from the control group, but a dose-dependent effect on the serum follistatin level was detected (figure 2B). These results indicate that the injected FST288 enters the systemic circulation. The possibility of any major degradation of the protein while in circulation was ruled out based on the absence of smaller protein bands in the serum western blot. Consistent with previous studies that have described the presence of one single isoform, FST315, in the blood circulation^24^,^25^, only a single band was detected from the serum of the animals in the FST 0 group (figure 2A). However, two separate bands of endogenous and injected recombinant FST288 were difficult to detect in the serum of animals that received FST288. This result could be due to the identical mobility of His-tagged FST288 and endogenous follistatin315. Accordingly, western blot analysis of serum using the anti-His-tagged antibody could only detect FST288, whereas the signal from the serum of the FST 0 group was undetectable (figure 2C).

### Daily administration of FST288 causes a change in the body composition in a dose-dependent manner

**Figure 3:**
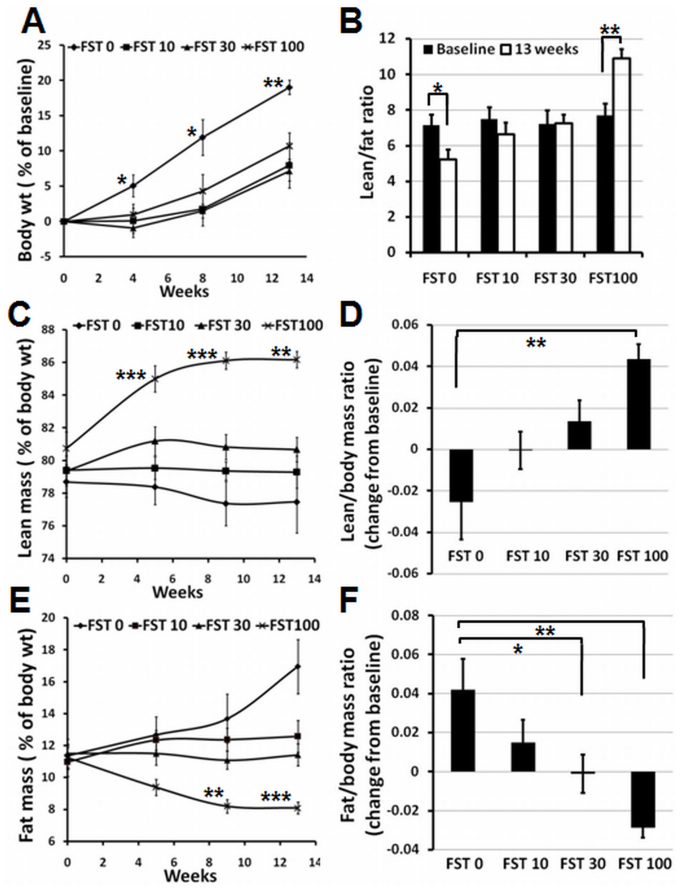
Growth and change in body composition in response to FST288 administration to mice. (A) Time course of the average body weight from all four groups, as indicated, represented as the percent of baseline. (B) Lean mass/fat mass ratio of animals, as indicated, after 13 weeks of treatment with FST288. (C & E) Time courses of lean mass and fat mass measurement, respectively, during the period of FST288 administration, plotted as the percent of body weight. (D & F) Lean mass/body mass and fat mass/body mass, respectively, of animals, as indicated, after 13 weeks of administration of FST288, plotted as the change (difference) from the baseline values. *, ** and *** indicate p < 0.05, <0.01 and <0.001, respectively. p-value measurements in A, C and E are between FST 0 and FST 100.

Body weight and body composition were assessed at the beginning of the experiments, and the values obtained were used as baseline for subsequent analyses. As shown in figure 3A, on average, there is approximately a 19% increase in the body weight during the course of 13 weeks in the control group (FST 0), which did not receive FST288. However, compared to the control group, body weight gain is significantly lower at the end of 13 weeks in all three groups that received FST288 (figure 3A). Among the three groups that received FST288, the FST 100 group showed the highest weight gain (10.7%). At the end of drug administration, the lean mass/fat mass ratio in the FST 0 group had significantly decreased from baseline, whereas in the FST 100 group, the ratio significantly increased (figure 3B).

The measurement of lean mass shows a progressive increase in the FST 100 group, whereas the FST 0 group shows a progressive decrease over time (figure 3C). After 13 weeks, compared to the FST 100 group, the lean mass content of the animals in the FST 0 group was lower than their baseline values, despite a significant increase in their body weight (figure 3A). The average baseline values were not significantly different among the four groups. There were no significant differences in the lean mass content measured from the 5^th^ week onwards between the FST 10 & 30 groups, in contrast to the FST 0 and FST 100 groups, which are significantly different (figure 3C). In fact, the ratio of the lean mass/body mass for FST 0 after 13 weeks is lower than the ratio at baseline, as observed in figure 3D. However, animals receiving FST288 showed an increase in the lean mass/body mass ratio, and a dose-dependent response was observed in all three experimental groups (figure 3D). In contrast to the lean mass, there was a progressive increase in the fat mass in the FST 0 group during the experiment, and interestingly, the fat mass in the FST 100 group was found to decrease following administration of FST288 (figure 3E). The decrease in the fat mass in the FST 100 group appeared significantly different from that in the FST 0 group from the 9^th^ week onwards; however, the fat mass of the FST 10 and 30 groups appeared significantly different from that of the FST 0 group only at the end of the 13^th^ week. Additionally, the measurement of fat mass/body mass ratio at the end of the course of administration demonstrated an increase in the amount of fat accumulation in the FST 0 group during the 13 week experiment but a decrease in the FST 100 group (figure 3F). The difference in the ratio between the FST 30 & 100 groups and the FST 0 group is statistically significant, and the average values indicate a dose-dependent effect on fat loss in the three experimental groups (figure 3F). A comparison of the ratio of lean mass/body mass and fat mass/body mass data (figure 3D & 3F) demonstrated that the administration of FST288 stimulates lean mass gain while reducing fat accumulation in the body at the same time in a dose-dependent manner.

### Postmortem dissection of tissues indicates muscle growth with decreased fat accumulation

**Figure 4:**
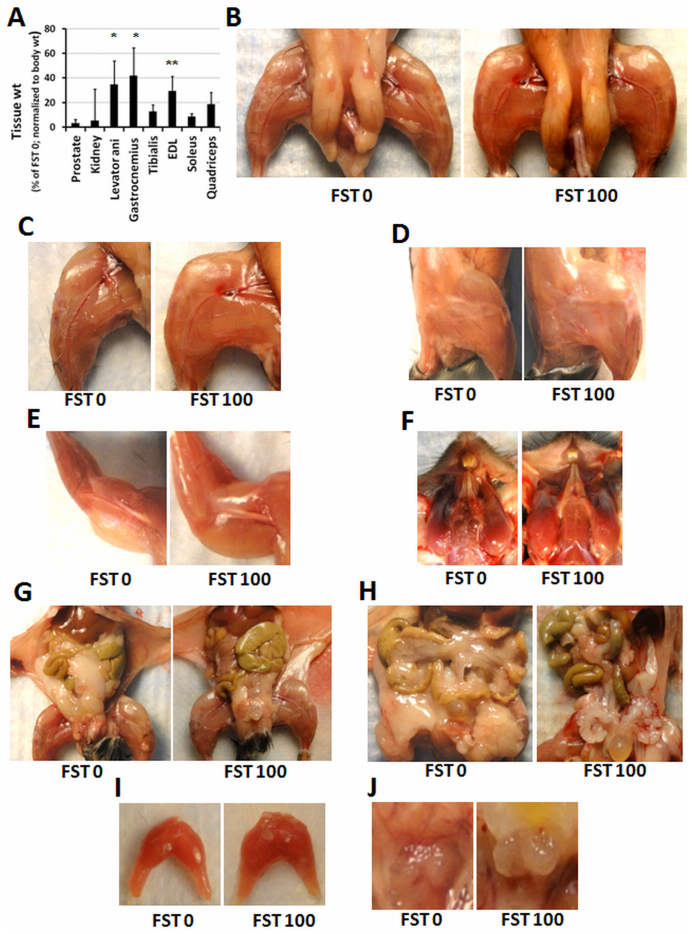
Post-mortem observation of muscles and organs. (A) Average weights of the dissected tissue, as indicated, of the FST 100 group presented as the percent of the FST 0 group (normalized to body weight). * and ** indicate p < 0.05 and <0.01, respectively. Pictures of lower limb (B, C and D), upper limb (E), facial (F), abdomen (G) abdominal visceral fat (H), dissected levator ani (I) and prostate (J) representing control (FST 0) and FST 100 groups, as indicated.

At the end of 13 weeks, FST288 administration was stopped, and the animals were euthanized. Individual tissues were removed and weighed and then preserved for further analyses. As mentioned earlier, animals receiving 100 μg/day FST288 showed the maximum effect of all tested doses on the body composition measurements; this result prompted the decision to analyze tissue specific changes between the FST 100 and FST 0 groups (control). Analysis of the individual muscle weights of the hind limbs of the animals demonstrated a significant increase in weight in the levator ani (approximately 34%), gastrocnemius (approximately 41%), and extensor digitorum longus muscle (EDL) (approximately 29%) in the FST 100 group compared to the control group (figure 4A). Additionally, a 8–18% weight increase in the tibialis anterior, soleus and quadriceps muscles in the FST 100 group was observed. Although weight gain was observed in the kidney and prostate tissues in the FST 100 group animals, the variability rendered the difference non-significant. The combined data indicate that there is an increase in the muscle mass in the FST 100-treated animals. The increase in the dissected muscle mass was validated by a visual comparison between the FST 100-treated mice and control mice (figure 4B–J). A comparison of the abdominal visceral fat accumulation in the FST 100 group shows a marked difference (figure 4) from the control animals, consistent with the fat mass data obtained by EchoMRI analysis.

### FST288 administration induces muscle fiber hypertrophy with myofiber remodeling

**Figure 5:**
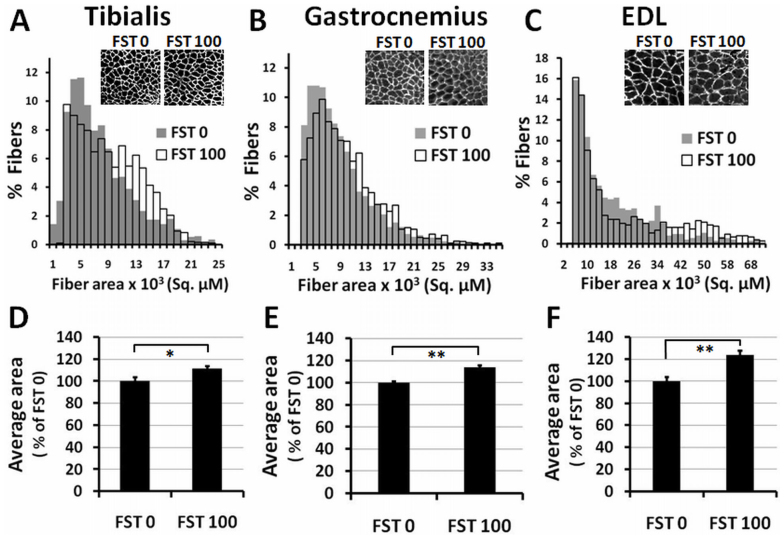
Muscle fiber area distribution of animals in the FST 0 and FST 100 groups. (A, B and C) Frequency distribution of areas from laminin stained muscle fibers, as indicated. (D, E and F) Average area of corresponding muscle fibers, plotted as a percent of the FST 0 group. Representative images of fiber area are presented in the inset. * and ** indicate p < 0.05 and <0.01, respectively.

To understand the anatomical changes in the muscle fiber organization, a histological analysis was performed on three isolated muscle tissues, namely, the gastrocnemius, tibialis anterior and EDL. Frozen cross-sections (8 μM) from the midbelly of each muscle were immunostained with the anti-laminin antibody, and the area within the laminin stained fibers was determined using Vision assistant software. Approximately two thousand fibers randomly selected from tissues belonging to the same group were analyzed, and the frequency distribution of the fiber area for all three muscles was plotted (figure 5A, B & C). Compared to the FST 0 group, the FST 100 animals demonstrated an increase in the average number of fibers with higher cross-sectional area in the distribution of all three muscles. Moreover, the average fiber area of the FST 100 group was significantly higher than that of the FST 0 group muscles (figure 5D, E & F) (approximately a 11%, 13% and 23% increase in the tibialis, gastrocnemius and EDL muscles, respectively). This observation indicates that there was hypertrophy of the muscle following FST288 treatment and that caused the increase in muscle mass. The total number of fibers was not significantly different in the control and FST 100 group muscles (tibialis and EDL) (data not presented).

**Figure 6:**
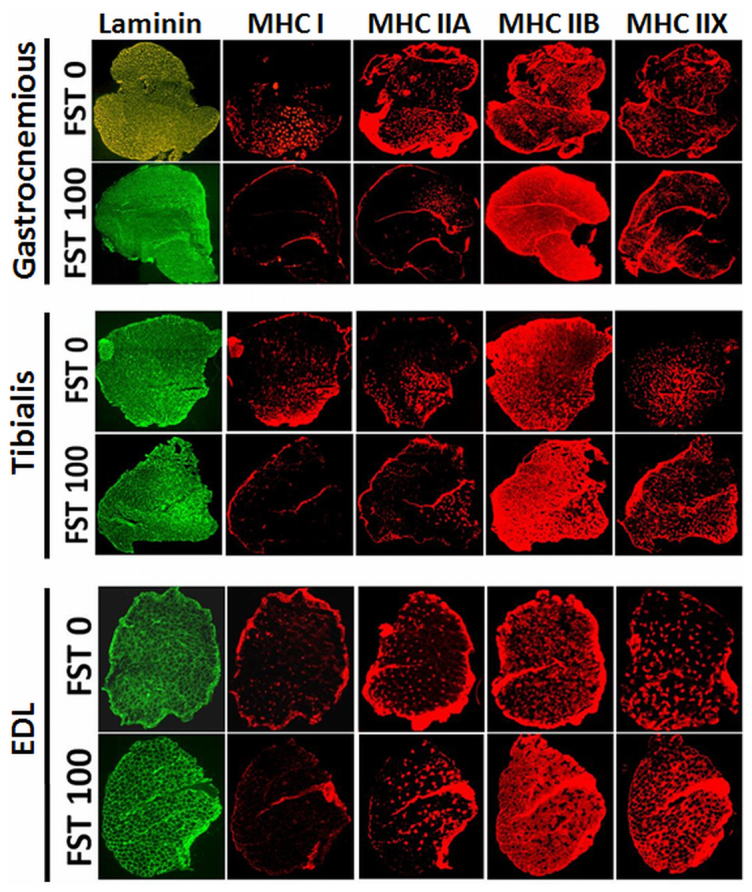
Analysis of muscle fiber types in muscles. Isoforms of the myosin heavy chains (MHC) stained with specific monoclonal antibodies (red) and co-stained with laminin (green), as indicated.

Skeletal muscle fibers are heterogeneous in terms of their size, fiber type and activity. This heterogeneity fulfills a variety of functions by a variety of muscles in the body; however, in response to changes in the metabolic activity and/or performance demand, skeletal muscles undergo remodeling of the fibers to meet functional requirements. To understand the changes in the myofiber type in the FST 100 treated animals, immunohistochemistry was performed using myosin heavy chain (MHC) isoform specific antibodies in the three muscles gastrocnemius, tibialis and EDL. Skeletal muscles are composed of four types of myosin heavy chain isoforms (type I and IIA, B & X), and isoform-specific mouse monoclonal antibodies were used for immunodetection. It should be noted that the isoform specific antibodies were raised in mice, thereby restricting the ability to co-stain a single muscle section for multiple isoforms. As a result, consecutive sections from the same muscle were stained with MHC I and IIA, B & X-specific monoclonal antibodies separately and co-stained with laminin and DAPI. As shown in figure 6, a change in the distribution of the MHC isoforms was observed in the muscles from the FST 100 group compared to the FST 0 group (see Supplementary figure S1–S3). Thus, the data suggest that in the gastrocnemius and tibialis, MHCI, MHCIIA & X are present in clusters in specific regions of the muscle, whereas MHCIIB is ubiquitous. However, EDL presents an almost homogenous distribution for all MHC isoforms tested. Careful observation indicates that compared to the FST 0 group, the number of MHC I & IIA fibers decreased and the number of MHCIIB fibers increased in all three muscles tested in the FST 100 group. However, the numbers of MHCIIX fibers either increased or were not affected by FST 100 administration.

### FST288 administration alters the metabolic rate and activity of mice

**Figure 7:**
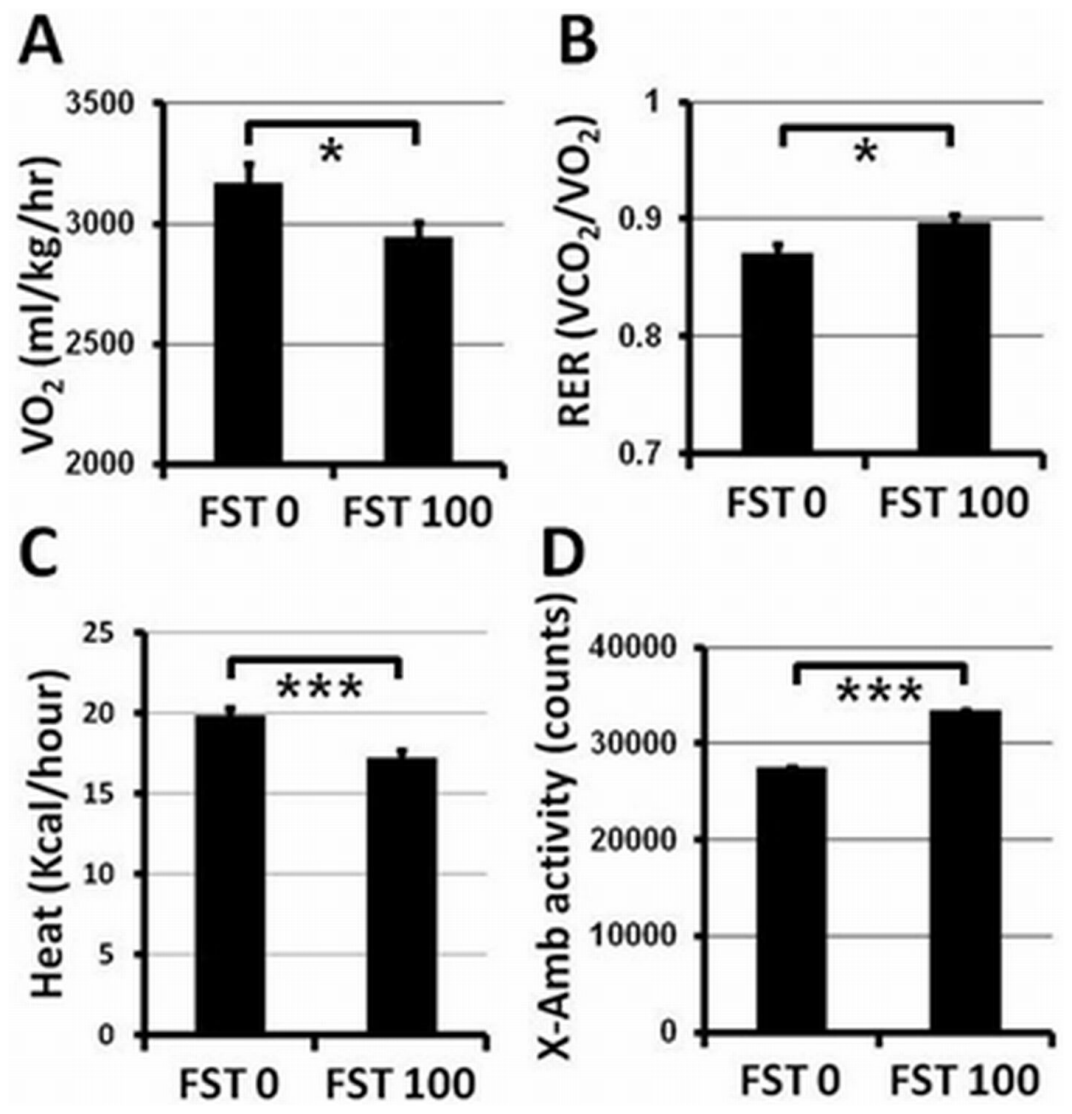
Metabolic measurements of the FST 0 and FST 100 group animals. Comparison of oxygen consumption (VO_2_) (A) respiratory exchange ratio (RER) (B) energy expenditure (heat) by indirect calorimetry (C) and X-axis ambulatory activity (D) between the FST 0 and 100 groups at the end of 13 weeks of FST288 administration. * and *** indicate p < 0.05 and <0.001, respectively.

To understand changes in the physiochemical status and activity of animals that result from FST288 treatment, metabolic measurements were performed on four animals from each of the FST 0 & 100 groups. The volume of oxygen consumption (VO_2_) and the volume of carbon dioxide (VCO_2_) production were calculated based on the fraction of gas passing through the input and at the exhaust point along with the air flow through the cage. An integrated program conducted the experimental operation and data collection. The data analysis indicated that the VO_2_ of the FST 100 group was significantly lower than that of the control animals (figure 7A). The respiratory exchange ratio (RER) is an indicator of metabolism that describes which fuel (carbohydrate or fat) is utilized to supply the body with energy, and it is calculated as the ratio of the volume of CO_2_ produced over the volume of O_2_ consumed (VCO_2_/VO_2_). The RER value is 1.0 when only carbohydrates are utilized, and the ratio is 0.7 when fat is predominantly utilized as the fuel source to supply the body with energy. The average RER value for the FST 100 group animals was significantly increased compared to that for the FST 0 group animals, as shown in figure 7B. This observation indicates that after 13 weeks of administration, the FST 100 group animals use glycolytic metabolism more than oxidative metabolism, which is consistent with the observed decrease in fat mass in the body. Total body energy expenditure is calculated as the equivalent of heat produced, which is determined from the RER and O_2_ consumption values by a method known as indirect calorimetry. These data indicate that the energy expenditure of the FST 100 group animals was significantly lower (approximately 15%) than that of the control animals (figure 7C). To correlate the metabolic rate with physical activity, spontaneous locomotor activity measurements (X-axis ambulatory activity) were performed. As shown in figure 7D, the X-axis ambulatory activity of the FST 100 animals was significantly higher (approximately 21% higher) than that of the control animals.

## Discussion

The present study describes the expression of the protein follistatin 288 (FST288) in *E. coli*. This protein was found to be biologically active and enters the systemic circulation after subcutaneous injection. The increased amount of FST288 in the serum of animals receiving a higher dose of FST288 indicated that the exogenous recombinant protein level in the serum can be altered by varying the amount of protein administered and that, more importantly, the injected FST288 circulated in the body without undergoing degradation (as revealed by gel electrophoresis mobility) (figure 1 and 2).

Many physiological responses resulted from FST288 administration. First, a progressive effect was observed over the course of administration. However, continuous monitoring of the mice indicated that the weight gain by the control group (which did not received FST288) was greater than that of the experimental groups. The lean mass/fat mass ratio became progressively lower for the control group (FST 0) over the course of the experiment, whereas this ratio increased in a dose-dependent manner for the experimental groups (Figure 3). More specifically, the data indicate that the loss of fat mass is associated with muscle mass gain.

The function of follistatin as a stimulator of muscle mass has been established in transgenic animals, but the current study indicates that the muscle mass gain is also associated with a concomitant fat loss. The fat loss was revealed by EchoMRI measurements, in addition to the visual difference in the visceral fat accumulation between the control and the FST 100 group animals. Furthermore, this study clearly indicates that follistatin is also a regulator of fat metabolism. The increase in muscle mass is possibly due to the increase in the average muscle fiber size (figure 5). Additionally, the data suggest that follistatin treatment modifies the muscle contraction machinery at the molecular level. Skeletal muscle is composed of four types of muscle fibers^26^,^27^,^28^, which can be distinguished based on the expression of four MHC isoforms (MHC I, IIA, IIB and IIX)^29^,^30^. Type I and IIA fibers are employed for slow twitches, whereas type IIB and IIX fibers are utilized for the fast movement of the muscle. The ratio of the four types of fibers present in the skeletal muscle varies in the muscle groups present in the body. Furthermore, the dynamic nature of the fiber types is due to alteration of their relative ratios in response to muscle activation, such as exercise, electrical stimulation, or in response to hormonal stimulation. MHC isoform types have been reported to switch in skeletal muscles depending on the physical demands^29^,^31^, and the remodeling of MHC compositions has been established to occur, by multiple experimental approaches, although the underlying mechanism is not yet clear. A combination of *in vivo* and *in vitro* studies indicates that intracellular calcium release in response to motor neuron stimulation plays a vital role in regulating calcineurin activity. Slow twitch fibers maintain a higher level of calcium, causing increased activation of calcineurin than fast twitch fibers, which, in turn, dephosphorylates transcription factors of the NFAT (nuclear factor of activated T cells) family. Dephosphorylated NFAT translocates to the nucleus and upregulates slow fiber specific gene expression. By contrast, decreased activation of calcineurin promotes the transition of slow-to-fast myofibers^31^,^32^.

Metabolite utilization also varies depending on the types of fibers present in the muscle. Type I and type IIA muscle fibers (slow twitch, high endurance) utilize oxidative metabolism, whereas type IIB and IIX fibers (fast twitch, low endurance) have a preference for glycolytic (anaerobic) metabolism^26^,^27^,^29^. The observation that the number of type IIB (also possibly IIX) fibers has increased and the numbers of type I and IIA fibers has decreased by the end of the current experiment defines a direct relationship with the increased RER values. This result further indicates that when the fat mass significantly decreases, glycolytic metabolism (utilization of carbohydrate) is preferred (figure 6 and 7). The overall metabolic activity measurement suggests that FST288 administration may promote high power/speed/force production for short sprints. The increase in muscle mass, as observed from the selected dose used in the systemic administration of FST288, is not as robust as that observed in transgenic animal studies^18^. Moreover, optimization of the dose and duration of administration may improve the drug's efficacy.

FST288 was selected instead of the other follistatin isoform (FST315) because FST315 is a free circulating isoform that lacks the ability to bind to cell surface proteoglycans^24^,^25^. It will be interesting to determine whether FST315 has a similar *in vivo* effect to FST288 using the same methods as in this study, although the function of FST315 has also been tested in rodents and nonhuman primates by gene therapy approaches^22^,^33^. Thus, the isoform specific function of follistatin in the regulation of muscle and fat mass remains to be clarified. The histology of adipose tissue is of significant interest to understand FST mediated change in morphology as well as accumulation of lipid droplets in the adipocytes and future study will proceed to that direction.

The most important observation of the current study is the physiological response detected to the injected recombinant protein, which leads us one step closer to the therapeutic administration of follistatin. The use of steroids as a stimulator of muscle mass is common amongst athletes, but the associated adverse effects restricts steroid use for the treatment of muscle degenerative diseases as well as of age related mobility limitations^34^,^35^,^36^,^37^ While scientists are trying to develop Selective Androgen Receptor Modulators (SARMs), follistatin could be a new candidate for future therapeutic application. Moreover, systemic administration as utilized in the current study has several advantages for clinical applications. With a daily injection, the doses can be controlled, and the drug can be administered as needed. Thus, overall, the study opens up the possibility of testing FST288 as a therapeutic agent to reverse muscle wasting conditions and aids our understanding of the functional mechanism of follistatin.

## Methods

### Expression and purification of human FST288

Follistatin 288 (FST288, amino acid residue no. 30–317) cDNA was cloned into the Sal1/Xho1 site of pET30 and expressed with a His-tag in *E. coli* BL21(DE3) by induction with IPTG. The cells were then resuspended in buffer containing 100 mM Tris-HCl, pH 8.0, and 10 mM NaCl and were sonicated on ice. The insoluble portion of the lysed cell suspension was separated by centrifugation, and the pellet was solubilized with a buffer containing 50 mM Tris-HCl, pH 8.0, 8 M urea and 100 mM PMSF. His-tagged FST288 was purified with a HisPur cobalt spin column (Thermo scientific, Rockford, IL) according to the manufacturer's instructions. The purified protein, in elution buffer containing 8 M urea, was diluted (1:4) with 200 mM Tris-HCl, pH 10.0, and 2 mM DTT and incubated on ice for 4–5 hours. The diluted protein was dialyzed against Tris buffer (10 mM Tris-HCl, pH 8.0, and 1 mM NaCl) or PBS at 4°C, with several modifications. The purified protein was subsequently passed through Detoxi-Gel (Endotoxin Removal Gel), Thermo Scientific, to remove bacterial endotoxins and stored at −80°C with 15–20% glycerol.

### Activity assay of the recombinant FST288

The biological activity of the recombinant FST288 was assessed by testing its ability to neutralize the growth inhibitory effect of activin on mouse MPC-11 cells, as described previously by Phillips et al^23^. Briefly, 1000 viable cells were seeded per well in a 96-well plate in a volume of 0.1 ml DMEM supplemented with 10% fetal calf serum, Pen-Strep and 25 μM β-mercaptoethanol. Activin (Stemgent, MA) or recombinant FST288 in the presence of activin was added, as indicated, to the culture medium, and cells were allowed to grow for 48 hours. Finally, 0.25 μCi of [^3^H] thymidine (6.7 Ci/mmol) was added to each well, and the cells were incubated for another 24 hours. The incorporation of [^3^H] thymidine into the cells was measured with a Cerenkov counter.

### Animal experiments

Animal experiments were conducted in accordance with the guidelines established and approved by the Institutional Animal Care and Use Committee (IACUC) of Boston University. Eight weeks old C57BL/6 mice were obtained from Jackson Laboratory (Bar Harbor, Maine) and housed in the Laboratory Animal Science Center (LASC) of Boston University Medical Center. Animals were allowed to recover for one week in the animal facility prior to the start of the experiments.

The recombinant FST288 in PBS containing 20% glycerol was subcutaneously injected into each animal daily for 13 weeks in a volume of 100 μl for all doses. The experiment was performed on four groups of animals with six individuals per group. The four groups were FST 0, 10, 30 and 100, with a daily FST administration of 0, 10, 30 and 100 μg per animal, respectively.

### Body composition

The body composition was assessed by two methods: 1) NMR using the EchoMRI-700 instrument and 2) postmortem dissected tissue weights.

An EchoMRI-700 (Body Composition Analyzer) from Echo Medical System (Houston, TX) was used to determine the fat mass and lean mass on a weekly basis in conscious, un-anesthetized animals. The body weight of each animal was measured, and the animals were placed individually into a plastic holder with limited restraint. Each scan took approximately 1–2 min. For postmortem measurement of muscle and other tissue weights, the individual muscles/tissues from each animal were dissected at the end of the 13 week administration, and the average weight was presented.

### Muscle morphometry

Dissected muscle, frozen with OCT compound, was cut at the midbelly, and 8 μm sections were transferred to glass slides. The sections were subjected to immunohistochemistry for laminin (rabbit anti-laminin Ab-1 antibody from NeoMarkers, Fremont, CA) to outline the muscle fibers for the measurement of fiber areas. For fiber typing, myosin heavy chain specific monoclonal antibodies were obtained from Developmental Studies Hybridoma Bank, University of Iowa. Type I (BA-D5), type IIA (SC-71), type IIB (BF-F3)^38^ and type IIX (6H1) antibodies^39^ were used, along with laminin for co-staining. Nuclei were stained with DAPI, and the images were acquired using the Eclipse TE 2000-U fluorescence microscope (Nikon Instruments Inc., Melville, NY). The fiber area measurements were performed using the Vision assistant software (National Instruments, Astin, TX).

### Metabolic assessments

Indirect calorimetry was utilized to examine oxygen consumption (VO_2_) and carbon dioxide production (VCO_2_) of individual mice for 24 hours in the fed status using a comprehensive laboratory animal monitoring system (CLAMS) equipped with an Oxymax Open Circuit Calorimeter (Columbus Instruments, Columbus, OH). VO_2_ and VCO_2_ values were used to calculate the respiratory exchange ratio (RER) and energy expenditure (heat - kcal/hr). Spontaneous locomotor (ambulatory) activity was recorded at the same time with indirect calorimetry using the Opto M3 multi-channel activity monitor (Columbus Instruments, Columbus, OH). The data were analyzed using Oxymat for Windows (V4.2) software.

### Western blotting

From each animal, at 13 weeks of FST288 administration, 0.5 μl of serum was, electrophoresed on a 12% SDS-PAGE gel, and the separated proteins were transferred to a PVDF membrane. The blot was probed with an anti-FST-antibody (K-19) from Santa Cruz Biotechnology, CA. The His-tag was detected by probing the blot with an anti-His antibody from Cell Signaling, MA.

### Statistical analysis

Experimental calculations for the activity assay, body compositions and metabolic activity measurements were performed using Microsoft Excel software. Muscle fiber distribution was determined using Prism software (Graphpad). The body weight and body composition values obtained at the beginning of the experiment were considered a baseline for subsequent analyses. Body weight gain was represented as the percent of the respective baseline values, and lean mass or fat mass was presented as the percent of the body weight. Dissected tissue weights were presented as the percent of control tissue (FST 0) weights.

## Author Contributions

S.S.G. designed and performed the experiments, prepared figures and wrote the manuscript.

## Supplementary Material

Supplementary Information Supplementary information
