# Supplementary material for: Systemic administration of Follistatin288 increases muscle mass and reduces fat accumulation in mice
Source: Sci Rep. 2013 Aug 14;3:2441. doi: 10.1038/srep02441 (PMC3743061; doi:10.1038/srep02441)
Supplement: Supplementary Information [file srep02441-s1.pdf]

Supplementary information

Systemic administration of Follistatin288 increases muscle mass and reduces fat accumulation in mice

Samudra S Gangopadhyay*

Section of Endocrinology, Diabetes, and Nutrition

Department of Medicine

Boston University School of Medicine

670 Albany Street, Boston, MA 02118

*Current address:

Department of Urology, Boston Children's Hospital, 300 Longwood Avenue, Boston, MA 02115 and Department of Surgery, Harvard Medical School, Boston MA

Correspondence:
Samudra S Gangopadhyay
Department of Urology
Boston Children's Hospital
300 Longwood Avenue
Boston, MA 02115
Phone: 617-919-3186
Fax: 617-730-0248
E-mail: Samudra.Gangopadhyay@childrens.harvard.edu
samudrag1234@gmail.com

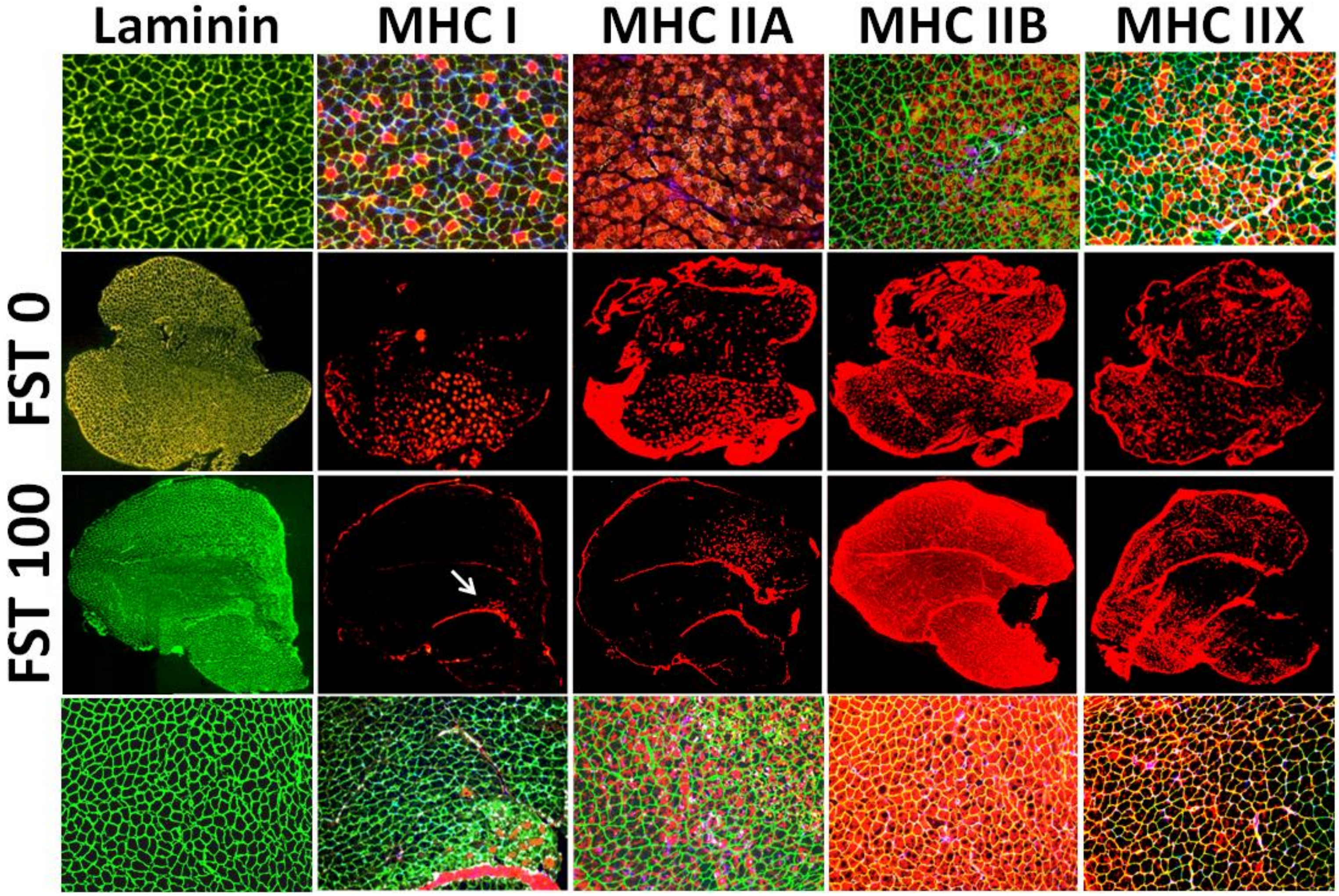

Supplementary figure S1. Isoforms of myosin heavy chains (MHC) stained with specific monoclonal antibodies (red) along with laminin staining (green) as indicated in Gastrocnemious. Higher magnification view from portions of the muscle co-stained for MHC (red), laminin (green) and DAPI (blue) are represented at the top or bottom corresponding to each muscle image.

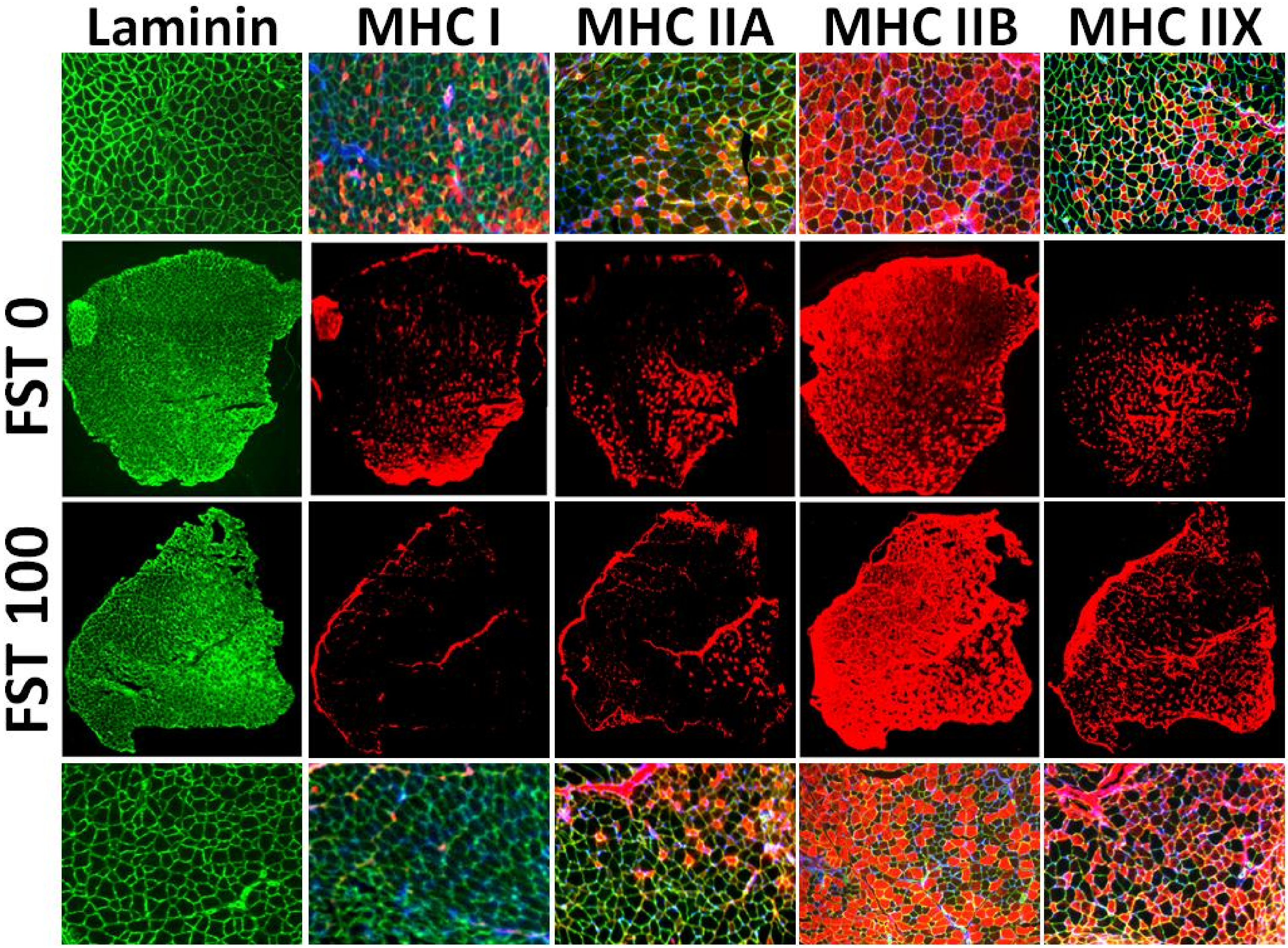

Supplementary figure S2. Isoforms of myosin heavy chains (MHC) stained with specific monoclonal antibodies (red) along with laminin staining (green) as indicated in Tibialis. Higher magnification view from portions of the muscle co-stained for MHC (red), laminin (green) and DAPI (blue) are represented at the top or bottom corresponding to each muscle image.

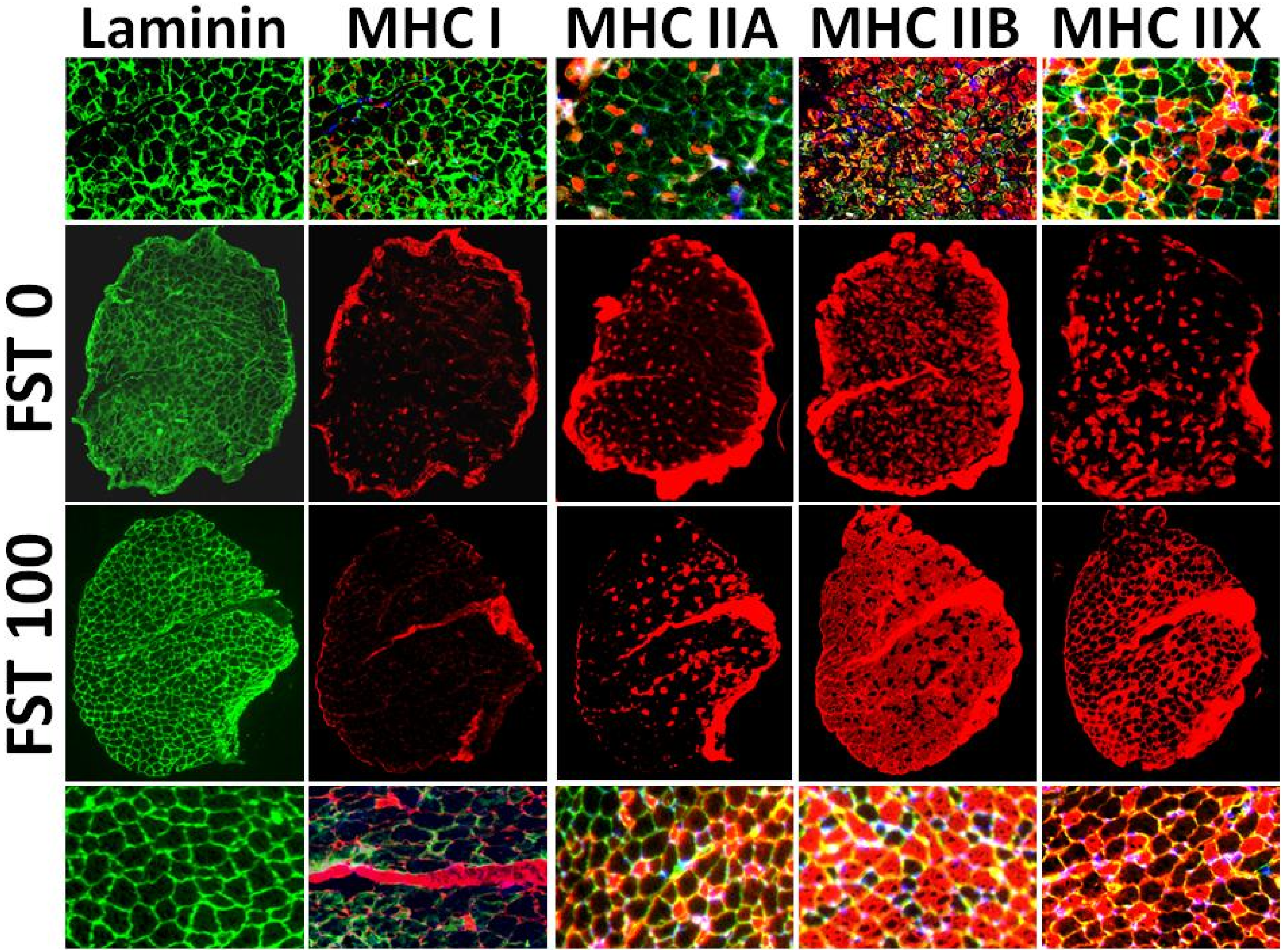

Supplementary figure S3. Isoforms of myosin heavy chains (MHC) stained with specific monoclonal antibodies (red) along with laminin staining (green) as indicated in Extensor Digitorum Longus (EDL). Higher magnification view from portions of the muscle co-stained for MHC (red), laminin (green) and DAPI (blue) are represented at the top or bottom corresponding to each muscle image.
